# Etymologia: Buruli Ulcer

**DOI:** 10.3201/eid2612.200744

**Published:** 2020-12

**Authors:** Tony M. Korman, Paul D.R. Johnson, John Hayman

**Affiliations:** Monash University, Melbourne, Victoria, Australia (T.M. Korman);; Austin Health, Melbourne (P.D.R. Johnson);; University of Melbourne, Melbourne (P.D.R. Johnson, J. Hayman)

**Keywords:** Buruli ulcer, Mycobacterium ulcerans, bacteria, tuberculosis and other mycobacteria, skin ulcers, cutaneous infections, Peter MacCallum, Uganda

**To the Editor:** The recent etymologia by Henry in the March 2020 issue of Emerging Infectious Diseases recounts the fascinating origin of the name Buruli ulcer (*1*). Further to the history, in 1948, pathologist Peter MacCallum first described the clinical features for 6 patients from Victoria, Australia, each with an ulcer with undermined edges on an arm or a leg, and the characteristic histopathologic findings, including extensive necrosis and abundant acid-fast bacilli without granuloma formation (*2*). Five of the patients were identified by general practitioners D.G. Alsop, L.E. Clay, and J.R. Searls from the city of Bairnsdale (thus, another eponym “Bairnsdale ulcer”) (*3*). Glen Buckle and Jean Tolhurst at the Alfred Hospital in Melbourne established experimental animal infections, and eventually isolated the causative organism (*2*), which they later named *Mycobacterium ulcerans* (*4*). The growth of *M. ulcerans* required prolonged incubation at a temperature of 30°C–33°C (*2*), which was only realized after the inadvertent use of a faulty incubator.

In 1964, Clancey described a “new” mycobacterium causing chronic skin ulcers in Uganda that “resembled” *M. ulcerans* which he named “*Mycobacterium buruli”* (*5*). However, the causative organism of Buruli ulcer was subsequently recognized as *Mycobacterium ulcerans,* which had been originally described in Australia.
